# Gammaherpesvirus infection modulates the temporal and spatial expression of SCGB1A1 (CCSP) and BPIFA1 (SPLUNC1) in the respiratory tract

**DOI:** 10.1038/labinvest.2014.162

**Published:** 2014-12-22

**Authors:** Gail H Leeming, Anja Kipar, David J Hughes, Lynne Bingle, Elaine Bennett, Nathifa A Moyo, Ralph A Tripp, Alison L Bigley, Colin D Bingle, Jeffery T Sample, James P Stewart

**Affiliations:** 1 Department of Infection Biology, University of Liverpool, Liverpool, UK; 2 Department of Veterinary Pathology, School of Veterinary Science, University of Liverpool, Liverpool, UK; 3 Institute of Veterinary Pathology, Vetsuisse Faculty, University of Zurich, Zurich, Switzerland; 4 Academic Unit of Oral and Maxillofacial Pathology, School of Clinical Dentistry, University of Sheffield, Sheffield, UK; 5 Department of Infectious Diseases, University of Georgia, Athens, GA, USA; 6 Investigative and Translational Pathology, AstraZeneca, R&D Innovative Medicines, Global Safety Assessment, Macclesfield, UK; 7 Academic Unit of Respiratory Medicine, Department of Infection and Immunity, University of Sheffield, Sheffield, UK; 8 Department of Microbiology and Immunology, Pennsylvania State University College of Medicine, Hershey, PA, USA

## Abstract

Murine *γ*-herpesvirus 68 (MHV-68) infection of *Mus musculus*-derived strains of mice is an established model of *γ*-herpesvirus infection. We have previously developed an alternative system using a natural host, the wood mouse (*Apodemus sylvaticus*), and shown that the MHV-68 M3 chemokine-binding protein contributes significantly to MHV-68 pathogenesis. Here we demonstrate in *A. sylvaticus* using high-density micro-arrays that M3 influences the expression of genes involved in the host response including *Scgb1a1* and *Bpifa1* that encode potential innate defense proteins secreted into the respiratory tract. Further analysis of MHV-68-infected animals showed that the levels of both protein and RNA for SCGB1A1 and BPIFA1 were decreased at day 7 post infection (p.i.) but increased at day 14 p.i. as compared with M3-deficient and mock-infected animals. The modulation of expression was most pronounced in bronchioles but was also present in the bronchi and trachea. Double staining using RNA *in situ* hybridization and immunohistology demonstrated that much of the BPIFA1 expression occurs in club cells along with SCGB1A1 and that BPIFA1 is stored within granules in these cells. The increase in SCGB1A1 and BPIFA1 expression at day 14 p.i. was associated with the differentiation of club cells into mucus-secreting cells. Our data highlight the role of club cells and the potential of SCGB1A1 and BPIFA1 as innate defense mediators during respiratory virus infection.

Murine *γ*-herpesvirus 68 (MHV-68, Murid herpesvirus 4 (MuHV-4)) infection of laboratory mice is a tractable and widely used small-animal model of *γ*-herpesvirus infection,^1, 2, 3, 4, 5, 6, 7^ specifically for the human pathogens Epstein–Barr virus and Kaposi's sarcoma-associated herpesvirus. Laboratory mice (*M. musculus*) are not a natural host for MHV-68.^8, 9, 10, 11^ So, to define host–pathogen interactions in an authentic system, we have developed infection of wood mice (*A. sylvaticus*), which are a natural host of MHV-68, as an alternative model.^12^ The host response to MHV-68 in wood mice differs from that seen in laboratory mice. Intra-nasal infection of wood mice with MHV-68 leads to productive replication in lung epithelial cells and in macrophages within granulomatous infiltrates that peaks at day 7 post infection (p.i.) and is resolved by day 21 p.i.^12^ This is followed by a latent infection of B cells in the spleen and in the lung within perivascular and peribronchiolar lymphocyte accumulations that leads to the formation of iBALT. The expansion of latently infected B cells peaks at day 14 p.i. but MHV-68 then establishes a latent infection in the spleen and lung that persists long term.^12^ The *M3* gene of MHV-68 encodes a viral chemokine-binding protein, known to bind a range of chemokines *in vitro*; however, its *in vivo* function could not be discerned when using laboratory mice that are a non-natural host.^13, 14^ Our previous experiments in wood mice using an MHV-68 mutant deficient in M3 showed that although M3 is not essential for infection, there is a marked alteration in the cellular response to the M3 mutant. Specifically, in wood mice infected with MHV-68 lacking M3, there is an alteration in the chemokine and cytokine environment, loss of the B-cell-dominated infiltrate in lungs at day 7 p.i., absence of iBALT formation at day 14 p.i., and a significantly reduced latent infection,^15^ further highlighting the significance of our wood mouse system.

The airway epithelium secretes multiple proteins that function in innate defense. Two highly expressed proteins that are thought to have this role are secretoglobin, family 1A, member 1 (SCGB1A1; also called uteroglobin, club (Clara) cell secretory protein or CC10) and BPI fold-containing family A1 (BPIFA1; also called SPLUNC1).^16, 17, 18, 19, 20^ SCGB1A1 is produced by non-ciliated epithelial, ie, club cells (Clara cells) in the airways.^16^ The precise role of SCGB1A1 has not been clearly defined and is likely to be multifactorial. However, *Scgb1a1*^−/−^ mice show both increased viral loads and increased pulmonary inflammation following respiratory syncytial virus infection^21^ and increased inflammatory, cytokine, and chemokine responses following infection with an E1- and E3-deleted adenoviral vector.^22^

BPIFA1 is found in the respiratory epithelium and submucosal glands of the upper airways and in the salivary glands in mice and humans.^17, 18, 19, 23, 24, 25^ The interspecies diversity and rapid evolution of BPIF genes^26, 27^ suggest a role for the proteins in host defense. BPIFA1 has a surfactant-like function^28^ and is involved in the regulation of the amiloride-sensitive epithelial sodium channel, ENaC.^29^ Infection with *Mycoplasma* spp. induces *Bpifa1* expression in murine airways^30^ and, importantly, BPIFA1 enhances IL-8 production and bacterial clearance.^30^ Recent data also suggest that the protein is important in the defense against *Klebsiella pneumoniae* infection^31^ and acts through modulation of macrophage function.^32^

As part of a study to identify transcriptional signatures associated with the MHV-68 M3 protein during infection, we identified a modulation of *Scgb1a1* and *Bpifa*1 expression. Here we describe the temporal and spatial changes in the expression of these two genes and their products during MHV-68 infection of wood mice.

## METHODS

### Cell Culture and Virus

Stocks of MHV-68, clone g2.4,^33^ and previously published, genetically engineered mutant MHV-68 viruses vM3.stop and vM3.MR^13^ were grown and titrated by infection of baby hamster kidney cells (BHK-21), as previously described.^34^ vM3.stop contains a stop codon inserted into the *M3* locus so as to disrupt the production of M3 protein. vM3.MR is a marker-rescue control virus derived from vM3.stop that expresses M3. BHK-21 cells were maintained in Glasgow's Modified Minimal Essential Medium with 10% newborn calf serum and 10% tryptose-phosphate broth, 2 mM L-glutamine, 70 *μ*g/ml penicillin, and 10 *μ*g/ml streptomycin.

### Wood Mice

Animal work was reviewed by the local University of Liverpool ethics committee and performed under UK Home Office Project Licence 40/2483 and personal licence 60/6501. MHV-68-negative, laboratory bred wood mice (*Apodemus sylvaticus*) were obtained from the established colony at the University of Liverpool, housed, and maintained as previously described.^12, 15^

### Virus Infection

Animals were randomly assigned into multiple cohorts, anesthetized lightly with halothane, and separate cohorts inoculated intra-nasally with either 4 × 10^5^ PFU of MHV-68 or recombinants vM3.stop (M3 deficient) or vM3.MR (marker-rescue control; kind gifts of Samuel Speck, Herbert W. Virgin IV and Victor van Berkel^13^) in 40 *μ*l sterile PBS, or were mock infected with PBS. They were killed on day 7 or 14 p.i. by cervical dislocation. Lungs and trachea were removed immediately and samples subjected to RNA purification or fixed for histology and transmission electron microscopy (TEM). A sample size of *n*=3 was used and was determined using power calculations and previous experience of experimental infection with these viruses. Separate cohorts of mice were used for microarray analysis, RNA (quantitative reverse transcriptase-PCR (qRT-PCR)) analysis, histopathology (RNA *in situ*, immunohistology (IH)), and electron microscopy.

### Microarray Analysis

Microarray work was performed by the University of Liverpool Centre for Genomic Research (http://www.liv.ac.uk/genomic-research/). Cohorts of three wood mice were infected with MHV-68 vM3.MR or vM3.stop^35^ and euthanized on day 14 p.i. Total cellular RNA was purified from the lungs using an RNeasy mini kit (Qiagen). We used one lung sample from each mouse per chip, ie, three biological replicates for each infection group. RNA quality was assessed using a 2100 bioanalyzer (Agilent Technologies) and 5 *μ*g used for array hybridization. RNA was reverse transcribed, amplified *in vitro*, biotinylated, cRNA fragmented, hybridized to Mouse Genome 430A V2 arrays, and scanned following Affymetrix protocols (http://www.affymetrix.com). Image processing and normalizations were performed using the Affymetrix MAS 5.0 software before input into GeneSpring (Agilent Technologies). The derived signal value was globally normalized and targeted to all probe sets equal to 100 before comparative analysis. Data were normalized per chip and to the 50th percentile. Signal intensities represent the abundance of RNA transcripts. Genes (probe sets) showing greater than twofold change in value and with a *P*<0.001 with respect to ‘Present' or ‘Absent' flags were chosen as changed genes. Genes whose expression was regulated more than twofold are described in Table 1.

### Cloning and Sequencing of Wood Mouse cDNAs

Total RNA was purified from lung tissue of uninfected *A. sylvaticus* using the RNeasy Mini Kit (Qiagen) and DNA contamination removed by treating RNA with amplification grade DNase I (Life Technologies) according to the manufacturers' recommendations. Reverse transcription was performed at 50 °C for 30 min with 2 *μ*g RNA in a 20-*μ*l reaction volume containing 200 U Superscript III reverse transcriptase (Invitrogen), 500 ng oligo(dT)_15_ primer (Roche), 0.5 mM dNTP mix (Promega), 5 mM DTT, 40 U RNase inhibitor (RNaseOUT; Life Technologies) in First-Strand buffer (50 mM Tris-HCl (pH 8.3), 75 mM KCl, 3 mM MgCl_2_; Life Technologies). Afterwards, 2 *μ*l was used as template for RT-PCR in 20 *μ*l reaction volumes. The oligodeoxynucleotide primers used for PCR are provided in Table 2. The cycling parameters were initially 95 °C for 10 min, and then for each cycle: 94 °C for 10 s, 60 °C for 20 s, and 72 °C for 60 s. The products were inserted into pCR2.1 using a TOPO-TA cloning kit (Life Technologies) according to the manufacturer's instructions. DNA from these cultures was extracted by alkaline lysis and sequenced commercially (Eurofins MWG, Germany). Six clones were sequenced for each gene and the sequence obtained compared with existing sequences using BLASTN.^36^ The sequences have been submitted to Genbank and have accession numbers HM008619 (*Scgb1a1*) and HM008620 (*Bpifa1*).

### Quantitative Reverse Transcriptase-PCR

qRT-PCR was performed as previously described^12^ using total RNA purified from the lungs (see above). Each sample was amplified in triplicate and the means from three animals were used and expressed relative to the copy number of the house-keeping gene 60S ribosomal protein L8 (*Rpl8)* cDNA. The oligodeoxynucleotide primers used for PCR are provided in Table 3.

### Histology, IH, and *In Situ* Hybridization

Lung and trachea were fixed in 4% buffered paraformaldehyde for 24–48 h and routinely paraffin wax embedded. Consecutive sections (3–5 *μ*m) were either stained with hematoxylin and eosin, used for IH or RNA *in situ* hybridization (RNA-ISH) and double stains.

IH was performed using the peroxidase anti-peroxidase method as previously described.^37, 38^ Primary antibodies used were rabbit anti-mSCGB1A1 (a kind gift of Barry Stripp)^39^ and rabbit anti-mBPIFA1 that was generated previously to an epitope localized in the N-terminal portion of the protein that is unique to the rodent lineage.^25^ The specificity of these targets had been determined previously. Papanicolaou's hematoxylin or the alcian blue/periodic acid–Schiff (AB-PAS) reaction for the demonstration of mucins were used as counterstains for the IH.

Detection of RNA by RNA-ISH followed a previously described protocol using digoxigenin-labeled sense and antisense probes, which were generated by *in vitro* transcription (digoxigenin RNA Labelling Kit (SP6/T7), Roche Applied Science, Mannheim, Germany).^12, 40, 41^ RNA-ISH probes were generated using IMAGE clones of mouse *Scgb1a1* (MGC:41130, IMAGE:1434396) and *Bpifa1* (MGC:62586, IMAGE:6314015) in pBluescript SK that were obtained via the Mammalian Gene Collection.^42^ Control sense strand probes were consistently found to show no reaction when hybridized to sections of wood mouse lung.

In addition, a combination of two techniques was used in which RNA-ISH was followed by IH.

### Quantitative Histopathological Assessment

The percentage area and density of DAB staining within airway epithelium was quantified using whole slide images scanned and analyzed using the Chromavision automated cellular imaging system (ACIS II) and ACIS Product version 2.4.8.0 (Clarient, Inc.) and Matrox® Imaging Library. Regions of interest comprising the airway epithelium and excluding other cells were outlined and defined as trachea, bronchus, or bronchiole. The ACIS system analyses thresholds of hue, luminosity, and saturation, which were set relative to the chromogen (DAB) utilized for BPIFA1 and SCGB1A1 localization. These data provided an average density of staining for each region of interest. The percentage area stained was defined as the brown area (positive immunostaining) divided by the total brown area plus blue area (hematoxylin; negative staining) × 100. The data from each anatomical area are presented as the mean±standard error of the mean (s.e.m.) for each time point of infection. Statistical significance was determined by Student's *t*-test and values of *P*<0.05 considered significant, calculated using Minitab v.15 (Minitab Inc.).

### Transmission Electron Microscopy

Sections of lung and trachea were fixed in 4% paraformaldehyde with 2.5% glutaraldehyde in 0.1 M sodium cacodylate buffer, and were routinely embedded in epoxy resin. Semi-thin sections (0.5 *μ*m) were prepared to select areas of interest. Ultrathin (60 nm) sections were cut, mounted on copper grids, stained with Reynold's lead citrate, and viewed with an H600 TEM (Hitachi).

### Statistical Analysis

Data were analyzed by one-way ANOVA with Bonferroni post-tests using the minitab v16 statistical package; *P*-values were set at 95% confidence interval.

## RESULTS

### Transcriptional Signatures Associated with MHV-68 M3 Expression During Infection

Our earlier study revealed that M3 is critical for modulating the host response wood mice. Thus, the efficient establishment of virus latency and formation of iBALT in the lung are dependent on M3 expression.^9, 15^ To gain further insight into the mechanism of M3 action, we identified genes that were differentially expressed in the presence and absence of M3. RNA extracted from the lungs of wood mice at day 14 p.i. with recombinant MHV-68 vM3.stop (M3-deficient) or vM3.MR (marker-rescue expressing M3) was analyzed using high-density microarrays. This revealed a limited number of genes (*n*=89) whose expression was at least twofold changed (Table 1). Functional annotation and grouping of these genes was performed. Of note were the obvious upregulation of genes encoding immunoglobulins and a downregulation of a high proportion of macrophage-specific genes in wood mice infected with vM3.MR as compared with vM3.stop infection. Of particular interest, several genes expressed in the airway epithelium encoding secreted glycoproteins were also upregulated in vM3.MR-infected wood mice. These included two members of the secretoglobin family (SCGB1A1 (club cell secretory protein or uteroglobin) and SCGB3A2 (uteroglobin-related protein 2)) and two members of the BPI fold-containing protein family (BPIFA1 (short PLUNC1, SPLUNC1) and BPIFB1 (long PLUNC1, LPLUNC1)). *Scgb1a1* and *Bpifa1* were highly differentially expressed (10.3- and 17.6-fold, respectively; Table 1 bold print). As it had been suggested that both these proteins were involved in airway defense,^19, 21^ we decided to analyze these proteins further during MHV-68 infection.

To validate the microarray results we performed qRT-PCR. However, to ensure accurate quantification, cDNAs corresponding to *Scgb1a1* and *Bpifa1* were generated from RNA extracted from wood mouse lung tissue using degenerate primers and cloned into pCR2.1. The DNA sequence of these clones revealed that the wood mouse *Scgb1a1*-coding sequence shares 93% identity at the nucleotide level with *M. musculus Scgb1a1* and that the wood mouse *Bpifa1*-coding sequence shares 94% identity at the nucleotide level with *M. musculus Bpifa1*. An alignment of the predicted amino-acid sequences of SCGB1A1 and BPIFA1 from wood mouse and other mammalian species was made using ClustalW2.0^43^ (Supplementary Figures E1 and E2). This revealed a high level of conservation of these proteins between wood mouse and house mouse. Of particular note is the conservation of an extended N-terminal glycine/proline-rich region between wood mouse and mouse BPIFA1 (Supplementary Figure E2) that has previously been shown to be rodent specific.

**Figure 1:**
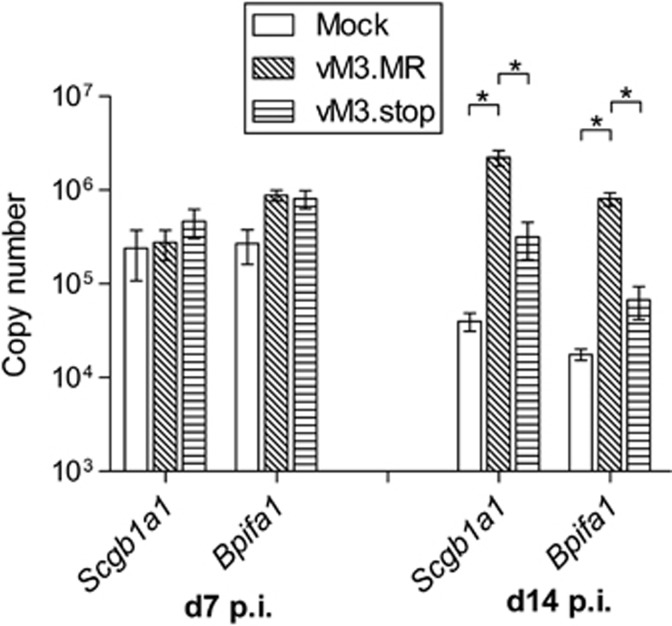
MHV-68 modulates the transcription of *Scgb1a1* and *Bpifa1* in the lungs of wood mice. RNA was extracted from the lungs of infected wood mice at days 7 and 14 p.i. as indicated and analyzed by qRT-PCR using primers specific for wood mouse *Bpifa1* and *Scgb1a1.* The copy number of mRNA was normalized to the copy number of cellular *Rpl8*. Bars represent mean±s.e.m. (*n*=3). Statistically significant differences (one-way ANOVA with Bonferroni post-tests) between groups are represented by square brackets above. *P<0.05.

To quantify *Scgb1a1* and *Bpifa1* mRNA, cohorts of wood mice were either mock-infected, infected with vM3.MR or with vM3.stop for either 7 or 14 days. These time points were chosen as we have shown previously that they correspond to the peak of productive virus replication and peak of latency expansion (and iBALT) in the wood mice.^12^ RNA was then extracted from the lung tissues and analyzed by qRT-PCR using wood mouse-specific primers (Table 3). At day 7 p.i., there were no significant differences in the levels of *Scgb1a1* and *Bpifa1* between any of the groups of wood mice (Figure 1). However, at day 14 p.i., the levels of both *Scgb1a1* and *Bpifa1* transcripts were significantly elevated (*P*<0.05) in the lungs of wood mice infected with vM3.MR as compared with both mock- and vM3.stop-infected animals. Both transcripts were also elevated in animals infected with vM3.stop as compared with mock-infected wood mice at this time point, although these changes were not statistically significant. This confirms our microarray data whereby expression of *Scgb1a1* and *Bpifa1* was elevated at day 14 p.i. in vM3.MR- as compared with vM3.stop-infected animals, but also show that there is a greater change when vM3.MR-infected are compared with mock-infected wood mice, suggesting that M3 was partially responsible for elevation of the expression of these host genes. Our previously published results^15^ have shown that vM3.stop is attenuated in wood mice, the viral load at day 14 p.i. being ca 1 log lower in M3.stop as compared with vM3.MR-infected animals. In addition, the chemokine and cytokine levels were shown in our previous study to vary between the two groups so it is equally possible that other factors are responsible for differences in SCGB1A1 and BPIFA1 expression. However, the modulation of SCGB1A1 and BPIFA1 expression does suggest a role for these factors during the viral infection. As these represented novel findings, and little is known about the function of these proteins in the context of viral infection, we decided to concentrate on how they behave during MHV-68 infection.

### SCGB1A1 Expression is Modulated after MHV-68 Infection

**Figure 2:**
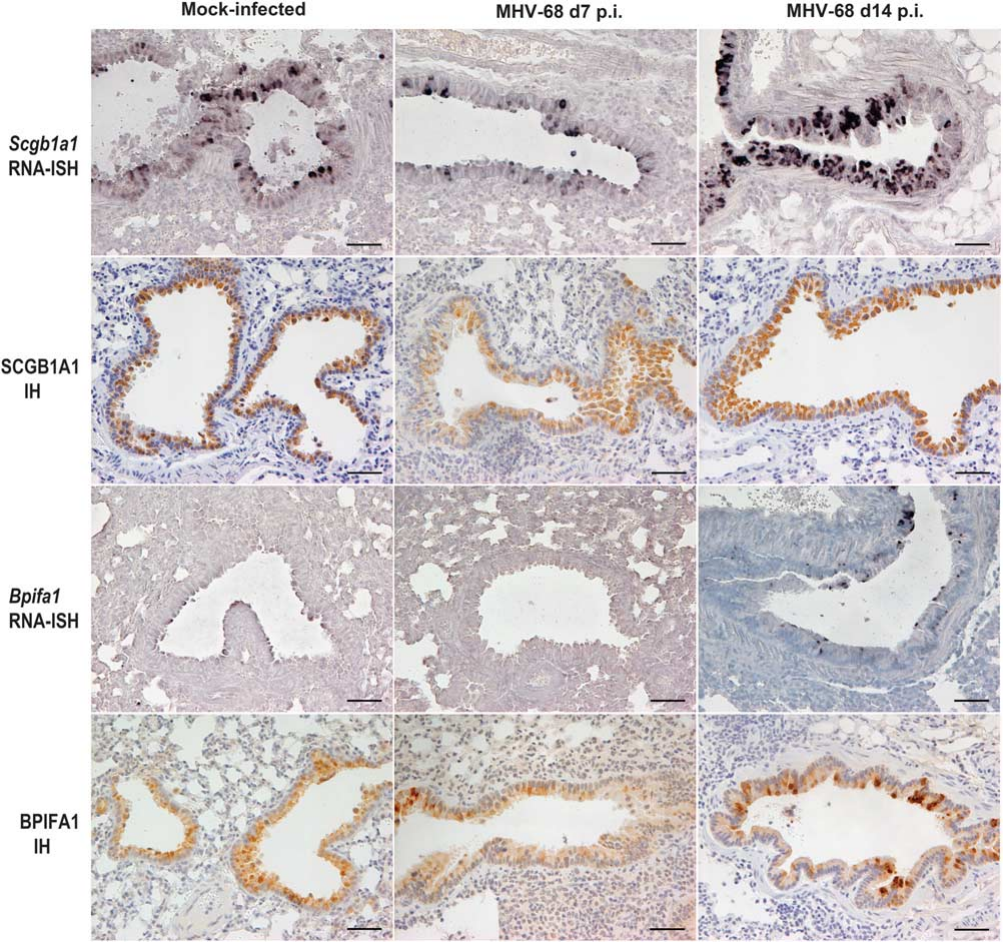
MHV-68 modulates the expression of SCGB1A1 and BPIFA1 RNA and antigen in bronchiolar epithelial cells. Lungs were harvested from wood mice that were either mock- or MHV-68-infected at either day 7 or day 14 p.i. as indicated. RNA specific for *Scgb1a1* and *Bpifa1* was detected by RNA-ISH using specific riboprobes, visualized with BCIP/NBT (dark blue–black), and counterstained with hematoxylin. Antigen was detected by immunohistology (IH) analysis using antibodies specific for SCGB1A1 and BPIFA1, visualized with DAB, and counterstained with hematoxylin. Scale bar represents 50 *μ*m.

To determine any anatomical- and/or temporal-based variation in SCGB1A1 after infection, trachea and lungs from wood mice that were either mock-infected or infected with MHV-68 were analyzed at day 7 and 14 p.i. (Figure 2).

Histopathological analysis of hematoxylin and eosin-stained sections revealed a pattern of changes after infection with MHV-68 consistent with our previous observations, represented predominantly by mutifocal mononuclear perivascular and peribronchiolar as well as multifocal granulomatous infiltration on day 7 p.i., and perivascular and peribronchiolar lymphocyte-dominated infiltration with iBALT formation at day 14 p.i. (not shown).

Expression of *Scgb1a1* was detected by RNA-ISH within mock-infected wood mice in rare epithelial cells in the trachea, but was seen more frequently in the bronchial epithelium. Cells in the larger proximal bronchioles stained most frequently for *Scgb1a1*, with numerous cells showing *Scgb1a1* transcripts. Smaller (terminal) bronchioles showed less frequent staining for *Scgb1a1* and the alveoli were negative. After infection with MHV-68, at both days 7 and 14 p.i. the trachea and bronchi showed a distribution of *Scgb1a1* expression similar to that observed in control wood mice (not shown). However, in the bronchioles, there was a downregulation of *Scgb1a1* expression at day 7 p.i. and an upregulation at day 14 p.i. (Figure 2, top row).

The presence of SCGB1A1 protein was determined by IH staining, which showed that the anatomical distribution of SCGB1A1 was similar to that of *Scgb1a1* RNA and to that previously described for *M. musculus*^44^ in both mock- and MHV-68-infected mice at both time points p.i. (Figure 2, second row).

**Figure 3:**
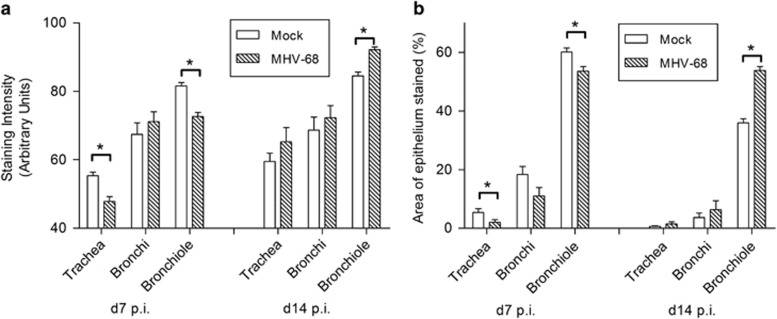
SCGB1A1 protein levels are modulated after MHV-68 infection. Lungs were harvested from wood mice that were either mock- or MHV-68-infected at either day 7 or day 14 p.i. as indicated. SCGB1A1 was detected by IH analysis using antibodies specific for SCGB1A1, visualized with DAB, and counterstained with hematoxylin. The intensity of staining (**a**) and percentage area of epithelium stained (**b**) in the trachea, bronchi, and bronchioles were assessed by image analysis. Data are for three mice per group presented as mean±s.e.m. (*n*=3). Significant differences from mock-infected mouse values are shown by an asterisk.

To assess further the amount of epithelial SCGB1A1 protein present across the respiratory tract, we performed quantitative analysis of the IH. At day 7 p.i., the intensity of staining for SCGB1A1 was significantly lower in the trachea (*P*<0.005) and bronchioles (*P*<0.05) in MHV-68-infected mice as compared with mock-infected control mice (Figure 3a). The percentage area of epithelium that stained positively for SCGB1A1 was similarly significantly decreased (Figure 3b). SCGB1A1 protein changes in the bronchi were not significant. At day 14 p.i., there were no significant alterations in the intensity of staining or the percentage of tissue stained in either the trachea or bronchi (Figure 3a and b), but within the bronchioles (closest to the site of viral replication) there were significant increases (*P*<0.005) in both parameters in MHV-68-infected wood mice compared with mock-infected wood mice (Figure 3).

### BPIFA1 Expression is Modulated after MHV-68 Infection

Anatomical and temporal changes in *Bpifa1* RNA and BPIFA1 protein expression following MHV-68 infection were similarly determined in the same wood mice used for SCGB1A1 analysis. The BPIFA1 antibody used was generated to an epitope localized in the N-terminal portion of the protein that is unique to the rodent lineage (Supplementary Figure E2). This epitope shares 13/16 amino acids with the mouse sequence used to generate the antibody.

In mock-infected wood mice, *Bpifa1* RNA was most notable in the trachea and bronchi, within the respiratory epithelium (non-ciliated cells; Figure 2, third row) and the submucosal glands. Within the bronchioles there were scattered *Bpifa1*-positive cells. We did not detect *Bpifa1* expression in the alveoli. Following infection with MHV-68, there were similar, high levels of *Bpifa1* transcription in the trachea and bronchi, and the proximal bronchioles showed an increase in transcription at day 14 p.i. (Figure 2, third row). The smaller terminal bronchioles were frequently negative, showing no changes from control animals.

IH analysis demonstrated that BPIFA1 protein was distributed in a similar pattern to the RNA, ie, within the non-ciliated epithelium of the trachea and bronchi and submucosal glands and to a lesser extent in the bronchiolar epithelium in both mock- and MHV-68-infected animals (Figure 2, bottom row).

**Figure 4:**
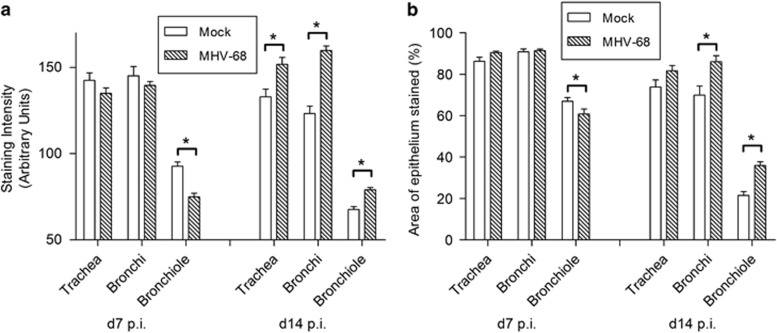
BPIFA1 protein levels are modulated after MHV-68 infection. Lungs were harvested from wood mice that were either mock- or MHV-68-infected at either day 7 or day 14 p.i. as indicated. BPIFA1 was detected by IH analysis using antibodies specific for BPIFA1, visualized with DAB, and counterstained with hematoxylin. The intensity of staining (**a**) and percentage area of epithelium stained (**b**) in the trachea, bronchi, and bronchioles were assessed by image analysis. Data are for three mice per group presented as mean±s.e.m. (*n*=3). Significant differences from mock-infected mouse values are shown by an asterisk.

Quantitative analysis (Figure 4) showed that although there was no significant difference in intensity of staining or percentage of tissue stained in the trachea and bronchi at day 7 p.i., there was a significant decrease in the intensity of staining (*P*<0.005) and in the percentage area stained (*P*<0.05) in the bronchioles at this time point. In contrast, at day 14 p.i., this pattern was reversed, as increases in BPIFA1 staining intensity was seen at all levels of the respiratory tract in MHV-68-infected compared with mock-infected animals (trachea *P*<0.05, bronchi and bronchioles *P*<0.05) and a significant increase in the area stained was seen in the bronchi and bronchioles. This increase did not correlate well with the staining for *Bpifa1* transcripts by RNA-ISH.

**Figure 5:**
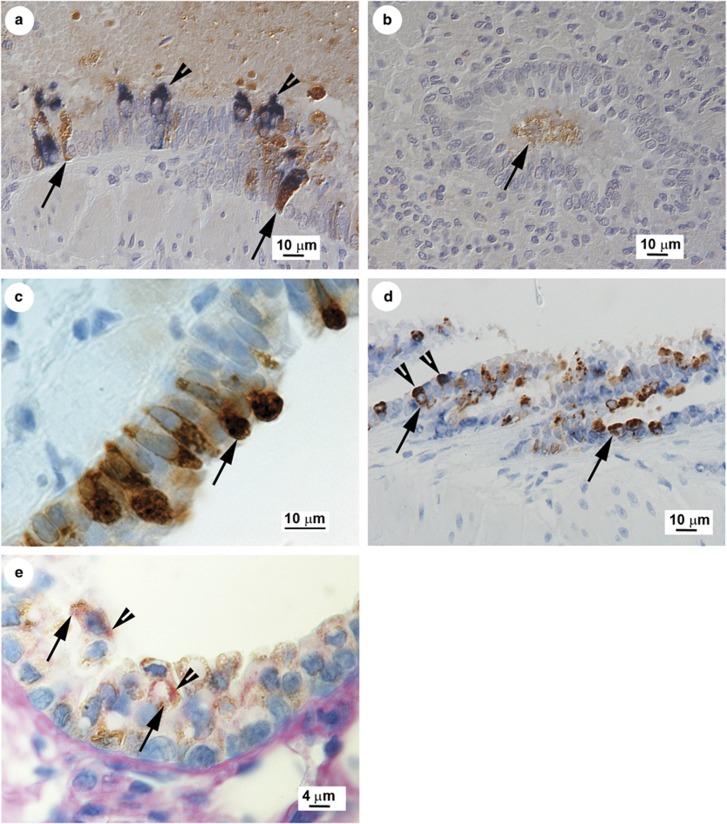
SCGB1A1 and BPIFA1 RNA and protein are co-expressed with mucus in club cells. (**a**, **b**) Combined IH analysis for BPIFA1 protein (visualized with DAB, arrow) and RNA-ISH with *Bpifa1-*specific probes (visualized with NBT/BCIP, arrowhead) demonstrates the presence of BPIFA1 protein in the distal terminal bronchioles (**b**) without evidence of *Bpifa1* transcription at this site, which occurred in the proximal bronchioles (**a**) and upper airways in MHV-68-infected wood mice at day 14 p.i. Hematoxylin counterstain. (**c**) IH analysis for BPIFA1 antigen (DAB, arrow) demonstrates granular staining in club cells in the cytoplasm consistent with intra-vesicular storage. (**d**) IH analysis for SCGB1A1 protein (DAB, arrows) was used to identify club cells and subsequent RNA-ISH with *Bpifa1-*specific probes demonstrated that club cells also transcribe *Bpifa1* (arrowheads). Hematoxylin counterstain. (**e**) Club cells identified by staining for SCGB1A1 protein (DAB, arrows) also contained AB-PAS-positive vesicles within the cytoplasm (arrowheads), consistent with mucous vesicles, suggesting that club cells also have the potential to produce mucus.

To investigate further the apparent discrepancy between the detection of *Bpifa1* RNA and BPIFA1 protein in bronchioles, double staining (IH and RNA-ISH) was performed on the same slide. The results showed co-localization of RNA and protein in trachea, bronchi, and proximal bronchioles (Figure 5a), but the presence of BPIFA1 protein without evidence of *Bpifa1* RNA in the terminal bronchioles (Figure 5b). In addition, BPIFA1 protein was localized predominantly intracellularly in larger airways (Figure 5a) within what appear to be storage granules (Figure 5c). In contrast, the strong staining observed in the terminal bronchioles was present not only within the airway epithelium but also in the airway lumen or apical surface of the cells (Figure 5b).

### Club Cells can be Multi-Functional

Club cells by definition secrete SCGB1A1.^45, 46^ To investigate if the same cells that produced SCGB1A1 also secreted BPIFA1, we used a combination of RNA-ISH and IH to determine co-localization. An RNA probe for *Bpifa1* was used followed by detection of SCGB1A1 by IH. This identified club cells in the bronchioles that expressed both *Bpifa1* transcripts and SCGB1A1 antigen (Figure 5d). To determine if club cells also produced mucins, IH using the SCGB1A1 antibody was performed as before and the AB-PAS reaction to highlight carbohydrates (neutral mucins: magenta, acidic mucins: blue) was applied as counterstain. The results (Figure 5e) identified club cells positive for SCGB1A1 that also contained AB-PAS-positive granules in the cytoplasm, consistent with mucus vesicles. Thus, we show for the first time that club cells producing SCGB1A1 not only can produce mucus after virus infection but also produce increased amounts of BPIFA1.

### Club Cells in the Bronchiole become Vesiculated after Infection

**Figure 6:**
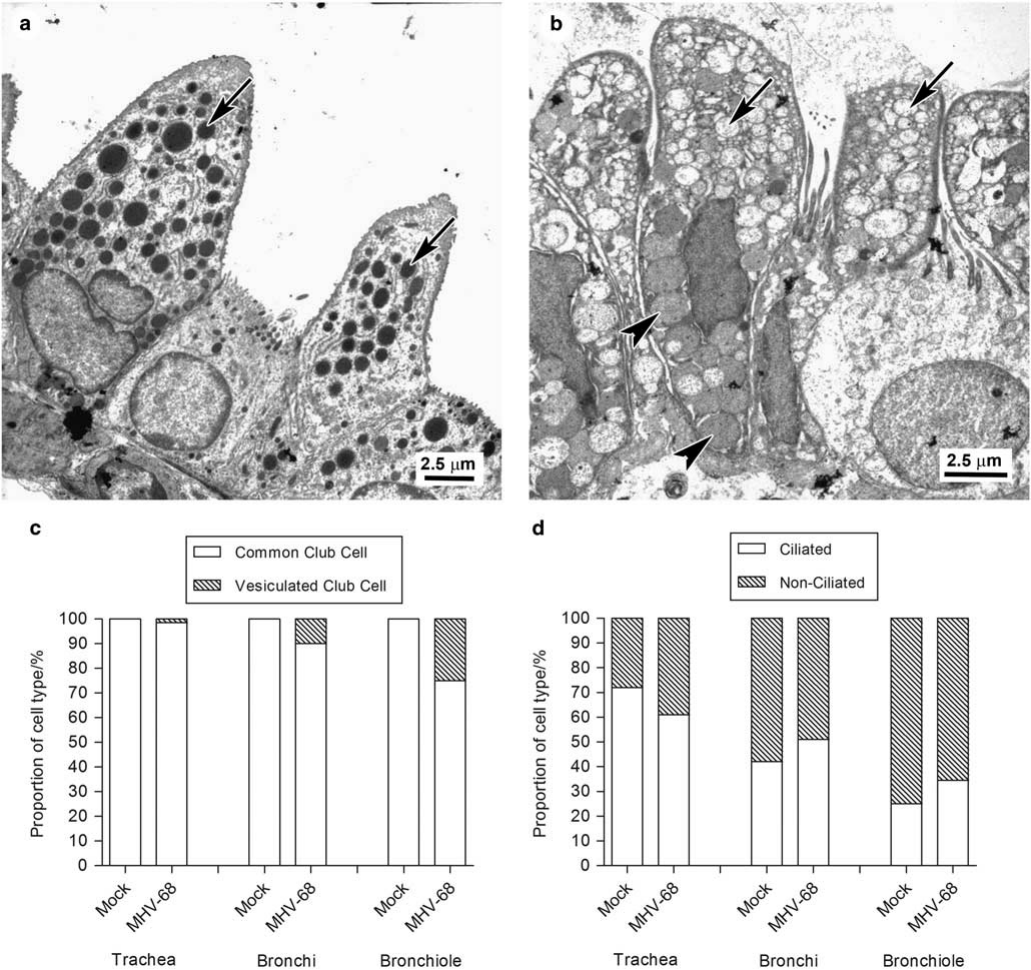
Transmission electron microscopic analysis of the morphology of club cells before and after infection. Transmission electron microscopy was used to quantify the morphological type and frequency of epithelial cells in the respiratory tract of wood mice. (**a**) Bronchiole. In mock-infected wood mice, the common type of club cell morphology vastly predominated with numerous electron-dense secretory vesicles in the apical part of the cell (arrow). (**b**) Bronchiole. A proportion of club cells, particularly in the bronchioles of MHV-68-infected wood mice at day 14 p.i. exhibited a distinct morphology with marked vesiculation of the apical part or all of the cytoplasm (arrows) and vesicles containing moderately electron-dense material characteristic of mucin (arrowheads). (**c**) The proportion of types of club cells in the airways was determined by enumeration of cell types present in electron micrograph images. (**d**) The proportion of ciliated and non-ciliated cells in the airways was determined by enumeration of cell types present in electron micrograph images.

As MHV-68 infection lead to an induction of mucus production by club cells, we wished to determine if this was associated with a corresponding alteration in their morphological phenotype. TEM was performed on lung tissue of mock- or MHV-68-infected wood mice at day 14 p.i. The proportion of ciliated *vs* non-ciliated epithelial cells was assessed as was the proportion of club cells with either a common or more vesiculated morphology indicative of mucus secretion, as previously defined.^44^ In mock-infected animals, the overwhelming majority of club cells demonstrated the typical or common morphology, including cytoplasm of moderate electron density, organized arrays of smooth endoplasmic reticulum, and electron-dense secretory vesicles in the apical part of the cell (Figure 6a and c) at all levels of the respiratory tract. In wood mice infected with MHV-68, in addition to the common type of club cell, vesiculated club cells were observed. These were infrequent in the trachea but increased in number in the distal airways (Figure 6b and c) making up ca 10% of bronchial and ca 25% of bronchiolar club cells. They exhibited marked vesiculation of the apical part or all of the cytoplasm and the vesicles contained moderately electron-dense material characteristic of mucin. There was no significant difference in the proportion of ciliated and non-ciliated epithelial cells revealed no significant difference after infection (Figure 6d).^44^

## DISCUSSION

We have developed MHV-68 infection of wood mice as an authentic system to study *γ*-herpesvirus infection. MHV-68 encodes a chemokine-binding protein termed M3, and in the current study, we used our wood mouse system along with a virus deficient in M3 expression (vM3.stop) to identify transcriptional signatures associated with the M3 protein during infection of the lung. Among other changes, this identified a significant upregulation of *Scgb1a1* and *Bpifa1* expression that was not only associated with expression of M3 but also with MHV-68 infection in general. We further examined the differential temporal and spatial expression of these two genes and their products in airways during MHV-68 infection of wood mice. This showed that BPIFA1 can be expressed and stored in granules within club cells along with SCGB1A1 and that there is a decrease in expression of both proteins in the bronchioles at day 7 p.i. consistent with release of stored protein. At day 14 p.i., the expression of both proteins increased. This is associated with the production of mucus in club cells.

Global analysis indicated differential expression of 89 genes between vM3.stop and vM3.MR-infected wood mice at day 14 p.i. (Table 1). Of note is the upregulation of a number of genes encoding immunoglobulins in mice infected with vM3.MR, which in likely a consequence of iBALT formation in these animals and its absence in vM3.stop-infected wood mice.^15^ It is also interesting that the expression of a high proportion of macrophage-specific genes was downregulated in vM3.MR-infected mice as compared with vM3.stop infection. This suggests disruption of macrophage function by M3 *in vivo* and fits well with the *in vitro* evidence for M3 binding to chemokines that affect macrophage function^13, 14^ and the *in vivo* evidence that M3 results in lower levels of macrophage-specific chemokines.^15^

The most highly differentially expressed genes at day 14 p.i. were *Scgb1a1* and *Bpifa1.* Differential expression was confirmed by qRT-PCR and in addition it was found that expression of both genes was lower in mock-infected animals than after either vM3.MR or vM3.stop virus infection (Figure 1). However, at day 7 p.i., there were no significant differences in the expression of either gene between any of the groups. A reduction in the level of expression of both genes in the mock control between day 7 and day 14 is likely due to alterations in expression caused by anesthesia and administration of PBS carrier and underlines the importance of using this control at both time points. The potential function of the M3 chemokine-binding protein in modulation of *Scgb1a1* and *Bpifa1* expression is interesting, but likely to be complex, and made more so by the M3-deficient virus being less pathogenic than vM3.MR, and hence, harboring a much higher viral load in the lung.^15^ Modulation of *Scgb1a1* and *Bpifa1* expression after MHV-68 infection clearly suggests a role for these factors in the host response. However, future studies will concentrate on if or how M3 might have a role in modulating expression of these interesting proteins.

The expression of *Scgb1a1* RNA was most frequently observed in the bronchiolar epithelium in mock-infected wood mice (Figure 2), but was also present throughout the respiratory epithelium, similar to that seen in *M. musculus.*^44^ At day 7 p.i. with MHV-68, there was a decrease in *Scgb1a1* RNA in the bronchioles and a significant decrease in both intensity of staining and percentage area stained for SCGB1A1 protein in the trachea and bronchioles (Figures 2 and 3). In contrast, there was no difference in expression of *Scgb1a1* mRNA detected by qRT-PCR between mock- and MHV-68-infected wood mice in the lungs at this time point (Figure 1). This discrepancy is probably due to the decreased RNA expression within bronchioles (as detected by RNA-ISH) being rendered undetectable by qRT-PCR analysis in a background of RNA extracted from whole lung tissue, including trachea and bronchi, where there was no difference in *Scgb1a1* RNA expression. This highlights the value of RNA-ISH in our analysis. However, at day 14 p.i., there was an increase in *Scgb1a1* expression in infected animals within the bronchiolar epithelium (Figures 1 and 2). In line with this, there were significant increases in SCGB1A1 protein in the bronchioles in MHV-68-infected wood mice (Figures 2 and 3).

The precise function of SCGB1A1 is not known, although it has been shown to inhibit phospholipase A2 and the production of IFN-*γ*, IL-1, and TNF-*α*.^16^ In addition, *Scgb1a1*^−/−^ mice infected with respiratory syncytial virus display an increased inflammatory response and also, increased viral titer when compared with wild-type animals.^21^ Mice deficient in SCGB1A1 that are infected with adenovirus also show a more intense inflammatory response.^22^ Thus, our observations of modulation of SCGB1A1 expression after infection with MHV-68 fit with a role in host defense and/or immunomodulation.

In laboratory mice, BPIFA1 is found predominantly in the upper respiratory tract and palate^47^ as a product of the airway epithelium^23, 47, 48^ and the submucosal glands.^48, 49^ In humans, it is found predominantly in the non-ciliated cells of the upper respiratory tract and submucosal glands.^50^ We observed a similar pattern of expression in wood mice (Figure 2). After infection, we detected *Bpifa1* RNA in non-ciliated epithelial cells in the trachea, bronchi, and proximal bronchioles but not terminal bronchioles (Figure 5a and b). When we analyzed BPIFA1 protein by IH, however, we found its presence in the entire respiratory tract, although the staining was less intense in the terminal bronchioles than in the trachea and bronchi (Figure 2), where the staining was mostly within granules in club cells (Figure 5c). Interestingly, IH analysis also identified the protein cell-free within the lumen of some terminal bronchioles (Figure 5b). Quantitative analysis showed that BPIFA1 levels were decreased in bronchioles at day 7 p.i. but increased throughout the entire respiratory tract in response to infection at day 14 p.i. (Figure 4). Our results show for the first time that BPIFA1 is produced and stored in club cells. The presence of BPIFA1 protein in the terminal bronchioles in the apparent absence of RNA is enigmatic. There is no precedence for secreted proteins to travel from higher up in the respiratory tract to terminal bronchioles, so BPIFA1 protein in this region could represent limited local production and rapid secretion from amounts of RNA that we did not detect by RNA-ISH. A recent report has described a decrease in BPIFA1 in the bronchio-alveolar lavage fluid after challenge of mice with bacteria and influenza A virus up to 7 days p.i. and suggested that BPIFA1 may act as a sensor of exposure to pathogens.^51^ The site of production of BPIFA1 was not analyzed in this study, nor were later time points, but the results concur with our observation of its reduced expression in bronchioles at day 7 p.i. during acute infection. In turn, the increased levels of BPIFA1 protein that we observed throughout the entire respiratory tract at day 14 p.i. may indicate a distinct role in the resolution of infection.

The cellular locations of SCGB1A1 and BPIFA1 overlap, but are not identical. We have shown here that cells positive for SCGB1A1 protein in bronchioles also transcribe *Bpifa1* (Figure 5d). Thus, we show for the first time that club cells are able to produce BPIFA1 and also appear to retain the protein within granules (Figure 5c). We also found the morphology of a proportion of club cells changed in response to infection and that the ‘vesiculated type' of club cell^44^ became much more frequent in the bronchioles of infected wood mice (Figure 6). The club cells also appeared to produce mucin following infection, as indicated by the AB-PAS reaction (Figure 5e). Club cells have been shown to produce mucus in response to antigen challenge,^52^ although other reports dispute that they have the capacity to store mucin and rather undergo metaplasia, becoming goblet-like cells.^53^ Boers *et al.*,^54^ in a survey of club cells in humans, found that SCGB1A1 expression and a positive PAS reaction within the same cell occurred in between 25 and 45% of goblet cells, depending on the location within the respiratory tract. This was defined as an ‘intermediate' cell type with characteristics of both goblet cells and club cells.^54^ Our results suggest that a proportion of club cells may undergo differentiation toward a goblet cell morphology, and may be in part the mechanism of mucous metaplasia observed in response to viral infection.^55^ The increase in BPIFA1 expression seen here at day 14 p.i. may also be associated with the phenotypic alteration of club cells into mucin-secreting cells.

Like SCGB1A1, the precise function of BPIFA1 is unknown. It has a surfactant-like effect^28^ and is involved in the regulation of the amiloride-sensitive epithelial sodium channel, ENaC.^29^ Infection with *Mycoplasma* spp. induces *Bpifa1* expression in murine airways^30^ and, importantly, BPIFA1 enhances IL-8 production and bacterial clearance.^30^ Recent data also suggest that the protein may be implicated in the mediation of host responses through modulation of macrophage function.^32^ Thus, the modulation of BPIFA1 expression after MHV-68 infection is consistent with a function in host defense and/or immune modulation including, possibly, sensing of infection.^51^

Our data show that the quantitative modulation of two proteins, SCGB1A1 and BPIFA1, is associated with MHV-68 infection. Although there are subtle differences, the temporal and spatial distribution of these two molecules throughout the respiratory tract is generally coordinated suggesting some commonality of regulation. Both proteins are stored in granules in club cells and their decrease in expression at day 7 p.i. is consistent with release of stored protein during the phase of acute MHV-68 virion production and epithelial cell damage. An increase in both proteins along with mucus and a change in morphology of a proportion of club cells likewise suggests a role for both proteins during the resolution of virion production and the establishment of latency. This also highlights a potentially important role of the club cell during virus infection in terms of secretion of factors involved in host defense.

## Figures and Tables

**Table 1:** Genes whose expression was modulated in the lungs of wood mice infected with vM3.MR compared with those infected with vM3.stop

Symbol	Description	Tissue/cell specificity	Biological process^a^	Ratio^b^
*Upregulated in vM3.MR-infected wood mice*				
			*Immune/inflammatory response*	
* Igha*	Immunoglobulin heavy constant *α*	Lymph node, intestine	Mucosal immune response	2.6
* Ighg1*	Immunoglobulin heavy constant gamma 1	Lymph node	Humoral immune response mediated by circulating Ig	4.5
* Ighg2b*	Immunoglobulin heavy constant gamma 2B	Lymph node	Humoral immune response mediated by circulating Ig	2.4
* Ighm*	Immunoglobulin heavy constant mu	Lymph node	Humoral immune response mediated by circulating Ig	2.8
* Ighm*	Immunoglobulin heavy constant mu	Lymph node	Humoral immune response mediated by circulating Ig	3
* Ighv1–72*	Immunoglobulin heavy variable 1–72	Lymph node, small intestine	Humoral immune response mediated by circulating Ig	2.7
* Igk-V8*	Immunoglobulin *κ* chain	B cells, lymph node	Humoral immune response mediated by circulating Ig	5.7
* Igkv8–30*	Immunoglobulin *κ* chain variable 8–30	B cells, lymph node	Humoral immune response mediated by circulating Ig	3.4
* Igkc*	Immunoglobulin *κ* constant	B cells, lymph node	B-cell differentiation	2.4
* Oas2*	2'-5' Oligoadenylate synthetase 2	Macrophage	Immune response/Rnase L induction	2
* C1qb*	Complement component 1, q subcomponent, *β* polypeptide	Macrophage, microglia, spleen, lymph nodes	Complement activation, classical pathway	2.1
* Itgb6*	Integrin *β* 6	Kidney, lung, skeletal muscle	Cell adhesion/signaling/inflammation	3.1
			*Cell signaling*	
* Dusp1*	Dual specificity phosphatase 1	Macrophage, lung, dendritic cell	Inactivation of MAPK activity	2
* Rem1*	Rad- and gem-related GTP-binding protein 1	Myoblasts	Signal transduction	2
* Map3k4*	Mitogen-activated protein kinase kinase kinase 4	Ubiquitous	Intracellular protein kinase cascade	4.8
* Tprkb*	Tp53rk-binding protein	liver, kidney, eye	protein catabolic process	2.3
* Ywhaq*	Tyrosine 3-monooxygenase/tryptophan 5-monooxygenase activation protein, theta polypeptide	Embryonal stem cells, neuronal tissue	Signal transduction	2
			*Regulation of expression*	
* Crx*	Cone-rod homeobox-containing gene	Retina	Regulation of transcription	3.5
* Myb*	Myeloblastosis oncogene	Myeloid progenitor, T cell	G1/S transition of mitotic cell cycle	2.2
* Bcas2*	Breast carcinoma amplified sequence 2	Embryonal stem cells	mRNA processing	2.3
* Paip1*	Polyadenylate-binding protein-interacting protein 1	stem cell, pro-B cell	Positive regulation of translation	7.4
			*Cell adhesion/morphogenesis*	
* Vcl*	Vinculin	Lung, mast cells	Focal adhesion plaque formation	3
* Cdh15*	Cadherin 15	Muscle	Cell adhesion	2.6
* Npnt*	Nephronectin	Lung, kidney	Cell–cell adhesion mediated by integrin	2.8
* Parva*	Parvin, *α*	Osteoblasts, stem cells, lung	Cell adhesion	2.9
			*Lung/respiratory tract*	
* Bpifa1*	**BPI fold-containing family A, member 1**	Lung, heart		**17.6**
* Bpifb1*	BPI fold-containing family B, member 1	Lung, stomach		7.3
* Scgb1a1*	**Secretoglobin, family 1A, member 1**	Lung		**10.3**
* Scgb3a2*	Secretoglobin, family 3A, member 2	Lung		3.2
			*Detoxification*	
* Cyp2f2*	Cytochrome P450, family 2, subfamily f, polypeptide 2	Lung, lacrimal gland, salivary gland, liver	Response to toxinoxidation-reduction process	2.2
* Cyp2a4*	Similar to cytochrome P450, family 2, subfamily a, polypeptide 4	Lacrimal gland	Oxidation-reduction process	12.8
			*Myeloid cell activation*	
* Snca*	Synuclein, *α*	Bone marrow, neuronal tissue	Myeloid leukocyte activation	2.1
* Trim10*	Tripartite motif-containing 10	Bone marrow	Myeloid cell haemopoiesis	4.5
			*Ion transport*	
* Slc4a1*	Solute carrier family 4 (anion exchanger), member 1	Bone marrow	Anion transport	2.2
* Lrrc26*	Leucine-rich repeat containing 26	Salivary gland, lacrimal gland	Ion transport	2
			*Miscellaneous*	
* Ap2a2*	Adaptor protein complex AP-2, *α* 2 subunit	CD4 T cell, granulocytes	Endocytosis	3
* Bc1*	Brain cytoplasmic RNA 1		Translational repressor activity	2.7
* Mus81*	MUS81 endonuclease homolog (yeast)	Adipose tissue, muscle	DNA repair	2.1
* Mccc2*	Methylcrotonoyl-Coenzyme A carboxylase 2 (*β*)	Adipose tissue, liver, kidney	Coenzyme A metabolic process	2
* Spa17*	Sperm autoantigenic protein 17	Testis	Ciliary or flagellar motility	2.1
* Myl4*	Myosin, light polypeptide 4	Heart, lung	Muscle contraction	2.5
* Agr2*	Anterior gradient homolog 2	Intestine, lacrymal gland	ER secreory pathway	4.2
* Agr3*	Anterior gradient homolog 3	Lung, testis	ER secreory pathway	10

*Downregulated in vM3.MR-infected wood mice*				
			*Regulation of expression*	
* Rnpc3*	RNA-binding region (RNP1, RRM) containing 3	Ubiquitous	Regulation of alternative mRNA splicing, via spliceosome	2.5
* Malat1*	Metastasis-associated lung adenocarcinoma transcript 1 (non-coding RNA)	Adrenal gland	Regulation of alternative mRNA splicing, via spliceosome	2.2
* Malat1*	Metastasis-associated lung adenocarcinoma transcript 1 (non-coding RNA)		Regulation of alternative mRNA splicing, via spliceosome	3.0
* Nsa2*	NSA2 ribosome biogenesis homolog	B cells, mast cells and **macrophages**	rRNA processing	4.3
* Ddx6*	DEAD (Asp-Glu-Ala-Asp) box polypeptide 6	B cells, T cells	Cytoplasmic mRNA processing body assembly	2.0
* C1d*	C1D nuclear receptor co-repressor	**Myeloid**, dendritic cells, osteoblasts	Regulation of transcription	2.9
* Mtf1*	Metal response element-binding transcription factor 1	Lacrimal gland, testis	Regulation of transcription	2.7
* Ankrd1*	Ankyrin repeat domain 1 (cardiac muscle)	Heart	Regulation of transcription	2.2
* Fubp1*	Far upstream element (FUSE)-binding protein 1	Embryonal stem cells, T cells, B cells	Regulation of transcription	6.0
* Nfic*	Nuclear factor I/C	Muscle, adipose tissue	Regulation of transcription	3.3
* Yap1*	Yes-associated protein 1	Embryonal Stem cells, placenta	Regulation of transcription	2.0
* Hist1h1c*	Histone cluster 1, H1c	Pancreas, intestine, osteoblast	Chromatin organization	4.0
* Srp9*	Signal recognition particle 9	Lacrimal gland, prostate	Negative regulation of translational elongation	3.0
			*Ion transport*	
* Slc39a8*	Solute carrier family 39 (metal ion transporter), member 8	Lung, uterus, cornea	Zinc ion transport	2.0
* Nipal2*	NIPA-like domain containing 2	Salivary, lacrimal glands	Magnesium ion transport	5.9
* Mfsd7c*	Major facilitator superfamily domain containing 7C	Placenta	Haeme transport	2.0
* Sln*	Sarcolipin	**Macrophage**, thymocyte	Regulation of calcium ion transport	7.3
* Atp6v0b*	ATPase, H+ transporting, lysosomal V0 subunit B	**Macrophage**, microglia	Proton transport	2.0
			*Cell signaling*	
* Rnd3*	Rho family GTPase 3	Dendritic cell, fibroblast, osteoblast	Small GTPase-mediated signal transduction	3.0
* Calm1*	Calmodulin 1	Ubiquitous	Response to calcium ion	7.0
* Leprot*	Leptin receptor overlapping transcript	Mast cell, **macrophage**, osteoblast	Negative regulation of JAK-STAT cascade	2.0
* Igbp1*	Immunoglobulin (CD79A)-binding protein 1	Granulocytes	Negative regulation of stress-activated MAPK cascade	2.4
* Camk1d*	Calcium/calmodulin-dependent protein kinase ID	Dendritic cells, neuronal tissue	Protein modification process	2.0
			*Immune/inflammatory response*	
* Igk-V1*	Immunoglobulin *κ* chain variable 1 (V1)	Intestine, spleen, B cell		2.4
* Bcap29*	B-cell receptor-associated protein 29	B cells, **macrophage**, testis	Apoptosis/intracellular transport	3.0
* Sod2*	Superoxide dismutase 2, mitochondrial	**Macrophage**, microglia	Response to reactive oxygen species	5.0
* Retnla*	Resistin like *α*	Adipose tissue, lung	Hormone activity/Inflammatory response	2.1
* Tbk1*	TANK-binding kinase 1	**Macrophage**	Positive regulation of interferon-*α*/*β*	2.0
			*Cell adhesion/morphogenesis*	
* Itgav*	Integrin *α* V	**Macrophage**, osteoblast	Cell adhesion/phagocytosis	4.8
* Sirpa*	Signal-regulatory protein *α*	**Macrophage**	Phagocytosis	2.5
* Sytl1*	Synaptotagmin-like 1; similar to synaptotagmin-like 1	Salivary, lacrimal glands	Exocytosis	4.7
* Cyfip1*	Cytoplasmic FMR1-interacting protein 1	**Macrophage**, microglia	Cell morphogenesis	6.8
			*Cell cycle*	
* Cdc123*	Cell division cycle 123	Ubiquitous	G1 phase of mitotic cell cycle	6.6
* Commd5*	COMM domain containing 5	**Macrophage**	Cell cycle arrest	2.2
			*Miscellaneous*	
* Zdhhc12*	zinc finger, DHHC domain containing 12	**macrophage**		3.0
* Ssfa2*	Sperm-specific antigen 2	epidermis, mast cells, **macrophage**		2.7
* Ndufa2*	NADH dehydrogenase (ubiquinone) 1 *α* subcomplex, 2	Ubiquitous	Electron transport chain	2.0
* Tpm3*	Tropomyosin 3, γ	NK cells, mast cells	Nervous system development	2.4
* Pgk1*	Phosphoglycerate kinase 1	**Macrophage**, skeletal muscle	Glucose catabolic process	3.8
* Bgn*	Biglycan	Osteoblast, fibroblast, lung	Extracellular matrix	11.7
* 2700089E24Rik*	RIKEN cDNA 2700089E24 gene	Salivary gland, mammary gland		4.3
* Ckap4*	Cytoskeleton-associated protein 4	Osteoblast, fibroblast		2.7
* Ttn*	Titin	Heart, mammary gland, skeletal muscle	Sarcomere organization	3.9
* Mb*	Myoglobin	Ubiquitous	Oxygen transport	2.7
* Acaa1a*	Acetyl-Coenzyme A acyltransferase 1A	Liver, kidney	Fatty acid metabolic process	4.2
* Hsd17b7*	Hydroxysteroid (17-*β*) dehydrogenase 7	Ovary, liver, **macrophage**	Steroid biosynthetic process	4.8

a Gene symbol, description, tissue/cell specificity, and Biological Process were annotated using DAVID (http://david.abcc.ncifcrf.gov/)^56, 57^ and BioGPS (http://biogps.org/).^58^

b Ratio of the normalized signal intensities obtained after Affymetrix genechip analysis for animals infected with vM3.MR *vs* vM3.stop. Only genes (probe sets) showing greater than twofold change in value and with a *P*<0.001 with respect to Present or Absent flags are represented.Bolded values are transcripts that were significantly upregulated and the focus of this manuscript.

**Table 2:** Primers used for amplification of *A. sylvaticus Scgb1a1* and *Bpifa1* cDNA for cloning

Primer	Gene	Sequence 5′→3′	Product length (bp)
SCGB1A1-f	*Scgb1a1*	CCTCTGGCCTCTACCATGAA	351
SCGB1A1-r	*Scgb1a1*	GACAGGGGCCTTTAGCAGTA	
BPIFA1-f	*Bpifa1*	ACTCAGACACCAAGAGAGAT	1011
BPIFA1-r	*Bpifa1*	CGTGAGGAGAAGGAAGACAT	

**Table 3:** Oligodeoxynucleotide primers used in quantitative RT-PCR

Primer	Gene	Sequence 5′→3′	Product length (bp)
RPL8int-f	*Rpl8*	ACAGAGCCGTTGTTGGTGTTGT	108
RPL8int-r	*Rpl8*	CAGTTCCTCTTTGCCTTGTACT	
bSCGB1A1-f	*Scgb1a1*	GATCGCCATCACAATCACTGTGG	156
bSCGB1A1-r	*Scgb1a1*	GTCTGAGCCAGGGTTGAAAGG	
bBPIFA1-f	*Bpifa1*	TGGCAGCCTGAAAATCAGCTTGC	161
bBPIFA1-r	*Bpifa1*	TGCACCAGGGTGACATCCAAAC	

## References

[bib1] Nash AA Dutia BM Stewart JP Natural history of murine gamma-herpesvirus infection Philos Trans R Soc Lond B Biol Sci 2001 356 569 579 11313012 10.1098/rstb.2000.0779 PMC1088445

[bib2] Speck SH Virgin HW Host and viral genetics of chronic infection: a mouse model of gamma-herpesvirus pathogenesis Curr Opin Microbiol 1999 2 403 409 10458986 10.1016/s1369-5274(99)80071-x

[bib3] Flano E Kim IJ Woodland DL gamma-herpesvirus latency is preferentially maintained in splenic germinal center and memory B cells J Exp Med 2002 196 1363 1372 12438427 10.1084/jem.20020890 PMC2193987

[bib4] Doherty PC Christensen JP Belz GT Dissecting the host response to a gamma-herpesvirus Philos Trans R Soc Lond B Biol Sci 2001 356 581 593 11313013 10.1098/rstb.2000.0786 PMC1088446

[bib5] Simas JP Efstathiou S Murine gammaherpesvirus 68: a model for the study of gammaherpesvirus pathogenesis Trends Microbiol 1998 6 276 282 9717216 10.1016/s0966-842x(98)01306-7

[bib6] Barton E Mandal P Speck SH Pathogenesis and host control of gammaherpesviruses: lessons from the mouse Annu Rev Immunol 2011 29 351 397 21219186 10.1146/annurev-immunol-072710-081639

[bib7] Wu TT Blackman MA Sun R Prospects of a novel vaccination strategy for human gamma-herpesviruses Immunol Res 2010 48 122 146 20717741 10.1007/s12026-010-8172-z PMC3931126

[bib8] Ehlers B Kuchler J Yasmum N Identification of novel rodent herpesviruses, including the first gammaherpesvirus of Mus musculus J Virol 2007 81 8091 8100 17507487 10.1128/JVI.00255-07 PMC1951306

[bib9] Hughes DJ Kipar A Milligan SG Characterization of a novel wood mouse virus related to murid herpesvirus 4 J Gen Virol 2010 91 867 879 19940063 10.1099/vir.0.017327-0 PMC2888160

[bib10] Hughes DJ Kipar A Leeming G Experimental infection of laboratory-bred bank voles (Myodes glareolus) with murid herpesvirus 4 Arch Virol 2012 157 2207 2212 22782137 10.1007/s00705-012-1397-5

[bib11] Blasdell K McCracken C Morris A The wood mouse is a natural host for Murid herpesvirus 4 J Gen Virol 2003 84 111 113 12533706 10.1099/vir.0.18731-0

[bib12] Hughes DJ Kipar A Sample JT Pathogenesis of a model gammaherpesvirus in a natural host J Virol 2010 84 3949 3961 20130062 10.1128/JVI.02085-09 PMC2849519

[bib13] van Berkel V Barrett J Tiffany HL Identification of a gammaherpesvirus selective chemokine binding protein that inhibits chemokine action J Virol 2000 74 6741 6747 10888612 10.1128/jvi.74.15.6741-6747.2000 PMC112190

[bib14] Parry CM Simas JP Smith VP A broad spectrum secreted chemokine binding protein encoded by a herpesvirus J Exp Med 2000 191 573 578 10662803 10.1084/jem.191.3.573 PMC2195820

[bib15] Hughes DJ Kipar A Leeming GH Chemokine binding protein M3 of murine gammaherpesvirus 68 modulates the host response to infection in a natural host PLoS Pathog 2011 7 e1001321 21445235 10.1371/journal.ppat.1001321 PMC3060169

[bib16] Singh G Katyal SL Clara cell proteins Ann N Y Acad Sci 2000 923 43 58 11193778 10.1111/j.1749-6632.2000.tb05518.x

[bib17] LeClair EE Four reasons to consider a novel class of innate immune molecules in the oral epithelium J Dent Res 2003 82 944 950 14630892 10.1177/154405910308201202

[bib18] Bingle CD Craven CJ PLUNC: a novel family of candidate host defense proteins expressed in the upper airways and nasopharynx Hum Mol Genet 2002 11 937 943 11971875 10.1093/hmg/11.8.937

[bib19] Bingle CD Gorr SU Host defense in oral and airway epithelia: chromosome 20 contributes a new protein family Int J Biochem Cell Biol 2004 36 2144 2152 15313462 10.1016/j.biocel.2004.05.002

[bib20] Broeckaert F Clippe A Knoops B Clara cell secretory protein (CC16): features as a peripheral lung biomarker Ann N Y Acad Sci 2000 923 68 77 11193780 10.1111/j.1749-6632.2000.tb05520.x

[bib21] Wang SZ Rosenberger CL Bao YX Clara cell secretory protein modulates lung inflammatory and immune responses to respiratory syncytial virus infection J Immunol 2003 171 1051 1060 12847279 10.4049/jimmunol.171.2.1051

[bib22] Harrod KS Mounday AD Stripp BR Clara cell secretory protein decreases lung inflammation after acute virus infection Am J Physiol 1998 275 L924 L930 9815110 10.1152/ajplung.1998.275.5.L924

[bib23] LeClair EE Nguyen L Bingle L Genomic organization of the mouse plunc gene and expression in the developing airways and thymus Biochem Biophys Res Commun 2001 284 792 797 11396972 10.1006/bbrc.2001.5024

[bib24] LeClair EE Nomellini V Bahena M Cloning and expression of a mouse member of the PLUNC protein family exclusively expressed in tongue epithelium Genomics 2004 83 658 666 15028288 10.1016/j.ygeno.2003.09.015

[bib25] Musa M Wilson K Sun L Differential localisation of BPIFA1 (SPLUNC1) and BPIFB1 (LPLUNC1) in the nasal and oral cavities of mice Cell Tissue Res 2012 350 455 464 22986921 10.1007/s00441-012-1490-9 PMC3505551

[bib26] Bingle CD LeClair EE Havard S Phylogenetic and evolutionary analysis of the PLUNC gene family Protein Sci 2004 13 422 430 14739326 10.1110/ps.03332704 PMC2286710

[bib27] Bingle CD Bingle L Craven CJ Distant cousins: genomic and sequence diversity within the BPI fold-containing (BPIF)/PLUNC protein family Biochem Soc Trans 2011 39 961 965 21787330 10.1042/BST0390961

[bib28] Gakhar L Bartlett JA Penterman J PLUNC is a novel airway surfactant protein with anti-biofilm activity PLoS One 2010 5 e9098 20161732 10.1371/journal.pone.0009098 PMC2817724

[bib29] Garcia-Caballero A Rasmussen JE Gaillard E SPLUNC1 regulates airway surface liquid volume by protecting ENaC from proteolytic cleavage Proc Natl Acad Sci USA 2009 106 11412 11417 19541605 10.1073/pnas.0903609106 PMC2708735

[bib30] Chu HW Thaikoottathil J Rino JG Function and regulation of SPLUNC1 protein in Mycoplasma infection and allergic inflammation J Immunol 2007 179 3995 4002 17785838 10.4049/jimmunol.179.6.3995

[bib31] Liu Y Bartlett JA Di ME SPLUNC1/BPIFA1 contributes to pulmonary host defense against Klebsiella pneumoniae respiratory infection Am J Pathol 2013 182 1519 1531 23499554 10.1016/j.ajpath.2013.01.050 PMC3644735

[bib32] Thaikoottathil JV Martin RJ Di PY SPLUNC1 deficiency enhances airway eosinophilic inflammation in mice Am J Respir Cell Mol Biol 2012 47 253 260 22499853 10.1165/rcmb.2012-0064OC PMC3423460

[bib33] Efstathiou S Ho YM Minson AC Cloning and molecular characterization of the murine herpesvirus 68 genome J Gen Virol 1990 71 1355 1364 2351958 10.1099/0022-1317-71-6-1355

[bib34] Sunil-Chandra NP Efstathiou S Arno J Virological and pathological features of mice infected with murine gamma-herpesvirus 68 J Gen Virol 1992 73 2347 2356 1328491 10.1099/0022-1317-73-9-2347

[bib35] van Berkel V Levine B Kapadia SB Critical role for a high-affinity chemokine-binding protein in gamma-herpesvirus-induced lethal meningitis J Clin Invest 2002 109 905 914 11927617 10.1172/JCI14358 PMC150927

[bib36] Altschul SF Madden TL Schaffer AA Gapped BLAST and PSI-BLAST: a new generation of protein database search programs Nucleic Acids Res 1997 25 3389 3402 9254694 10.1093/nar/25.17.3389 PMC146917

[bib37] Stewart JP Kipar A Cox H Induction of tachykinin production in airway epithelia in response to viral infection PLoS One 2008 3 e1673 18320026 10.1371/journal.pone.0001673 PMC2248620

[bib38] Kipar A Kohler K Leukert W A comparison of lymphatic tissues from cats with spontaneous feline infectious peritonitis (FIP), cats with FIP virus infection but no FIP, and cats with no infection J Comp Pathol 2001 125 182 191 11578135 10.1053/jcpa.2001.0501

[bib39] Chen H Matsumoto K Brockway BL Airway epithelial progenitors are region specific and show differential responses to bleomycin-induced lung injury Stem Cells 2012 30 1948 1960 22696116 10.1002/stem.1150 PMC4083019

[bib40] Terry LA Stewart JP Nash AA Murine gammaherpesvirus-68 infection of and persistence in the central nervous system J Gen Virol 2000 81 2635 2643 11038374 10.1099/0022-1317-81-11-2635

[bib41] Dutia BM Stewart JP Clayton RA Kinetic and phenotypic changes in murine lymphocytes infected with murine gammaherpesvirus-68 *in vitro* J Gen Virol 1999 80 2729 2736 10573167 10.1099/0022-1317-80-10-2729

[bib42] Strausberg RL Feingold EA Grouse LH Generation and initial analysis of more than 15,000 full-length human and mouse cDNA sequences Proc Natl Acad Sci USA 2002 99 16899 16903 12477932 10.1073/pnas.242603899 PMC139241

[bib43] Larkin MA Blackshields G Brown NP Clustal W and clustal X version 2.0 Bioinformatics 2007 23 2947 2948 17846036 10.1093/bioinformatics/btm404

[bib44] Pack RJ Al-Ugaily LH Morris G The cells of the tracheobronchial epithelium of the mouse: a quantitative light and electron microscope study J Anat 1981 132 71 84 7275793 PMC1233396

[bib45] Bedetti CD Singh J Singh G Ultrastructural localization of rat Clara cell 10 KD secretory protein by the immunogold technique using polyclonal and monoclonal antibodies J Histochem Cytochem 1987 35 789 794 2438324 10.1177/35.7.2438324

[bib46] Singh G Singh J Katyal SL Identification, cellular localization, isolation, and characterization of human Clara cell-specific 10 KD protein J Histochem Cytochem 1988 36 73 80 3275712 10.1177/36.1.3275712

[bib47] Weston WM LeClair EE Trzyna W Differential display identification of plunc, a novel gene expressed in embryonic palate, nasal epithelium, and adult lung J Biol Chem 1999 274 13698 13703 10224143 10.1074/jbc.274.19.13698

[bib48] Leclair EE Four BPI (bactericidal/permeability-increasing protein)-like genes expressed in the mouse nasal, oral, airway and digestive epithelia Biochem Soc Trans 2003 31 801 805 12887309 10.1042/bst0310801

[bib49] Di YP Harper R Zhao Y Molecular cloning and characterization of spurt, a human novel gene that is retinoic acid-inducible and encodes a secretory protein specific in upper respiratory tracts J Biol Chem 2003 278 1165 1173 12409287 10.1074/jbc.M210523200

[bib50] Bingle L Cross SS High AS SPLUNC1 (PLUNC) is expressed in glandular tissues of the respiratory tract and in lung tumours with a glandular phenotype J Pathol 2005 205 491 497 15685591 10.1002/path.1726

[bib51] Britto CJ Liu Q Curran DR Short palate, lung, and nasal epithelial clone-1 is a tightly regulated airway sensor in innate and adaptive immunity Am J Respir Cell Mol Biol 2013 48 717 724 23470624 10.1165/rcmb.2012-0072OC PMC3727874

[bib52] Evans CM Williams OW Tuvim MJ Mucin is produced by clara cells in the proximal airways of antigen-challenged mice Am J Respir Cell Mol Biol 2004 31 382 394 15191915 10.1165/rcmb.2004-0060OC PMC10862391

[bib53] Davis CW Dickey BF Regulated airway goblet cell mucin secretion Annu Rev Physiol 2008 70 487 512 17988208 10.1146/annurev.physiol.70.113006.100638

[bib54] Boers JE Ambergen AW Thunnissen FB Number and proliferation of clara cells in normal human airway epithelium Am J Respir Crit Care Med 1999 159 1585 1591 10228131 10.1164/ajrccm.159.5.9806044

[bib55] Walter MJ Morton JD Kajiwara N Viral induction of a chronic asthma phenotype and genetic segregation from the acute response J Clin Invest 2002 110 165 175 12122108 10.1172/JCI14345 PMC151043

[bib56] Huang da W Sherman BT Lempicki RA Systematic and integrative analysis of large gene lists using DAVID bioinformatics resources Nat Protoc 2009 4 44 57 19131956 10.1038/nprot.2008.211

[bib57] Huang da W Sherman BT Lempicki RA Bioinformatics enrichment tools: paths toward the comprehensive functional analysis of large gene lists Nucleic Acids Res 2009 37 1 13 19033363 10.1093/nar/gkn923 PMC2615629

[bib58] Wu C Orozco C Boyer J BioGPS: an extensible and customizable portal for querying and organizing gene annotation resources Genome Biol 2009 10 R130 19919682 10.1186/gb-2009-10-11-r130 PMC3091323

