# Supplementary material for: Gammaherpesvirus infection modulates the temporal and spatial expression of SCGB1A1 (CCSP) and BPIFA1 (SPLUNC1) in the respiratory tract
Source: Lab Invest. 2014 Dec 22;95(6):610–24. doi: 10.1038/labinvest.2014.162 (PMC4450743; doi:10.1038/labinvest.2014.162)
Supplement: Supplementary Information [file labinvest2014162x1.ppt]

## Slide 1
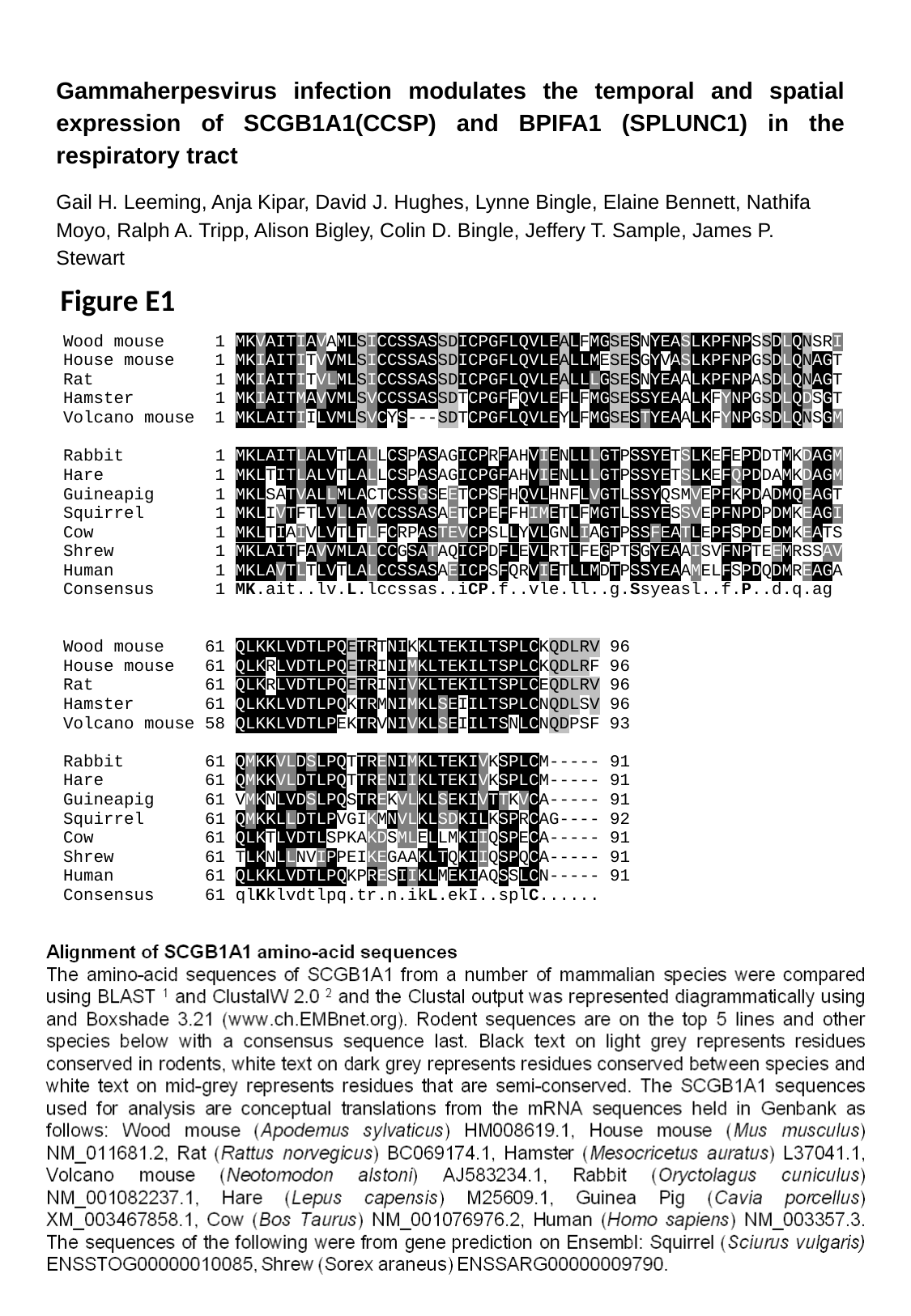

Gammaherpesvirus infection modulates the temporal and spatial expression of SCGB1A1(CCSP) and BPIFA1 (SPLUNC1) in the respiratory tract
Gail H. Leeming, Anja Kipar, David J. Hughes, Lynne Bingle, Elaine Bennett, Nathifa Moyo, Ralph A. Tripp, Alison Bigley, Colin D. Bingle, Jeffery T. Sample, James P. Stewart
Figure E1

## Slide 2
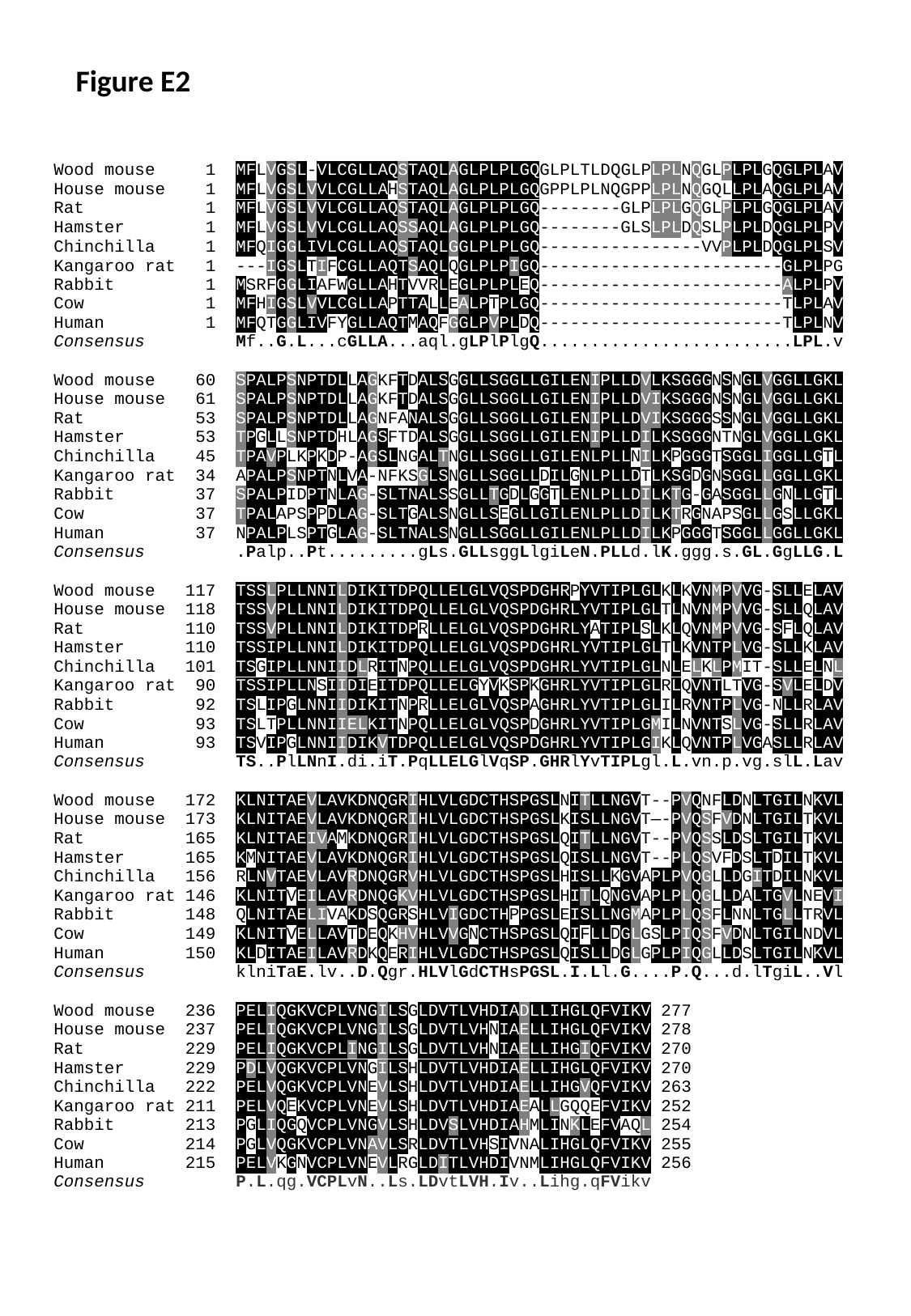

Figure E2

## Slide 3
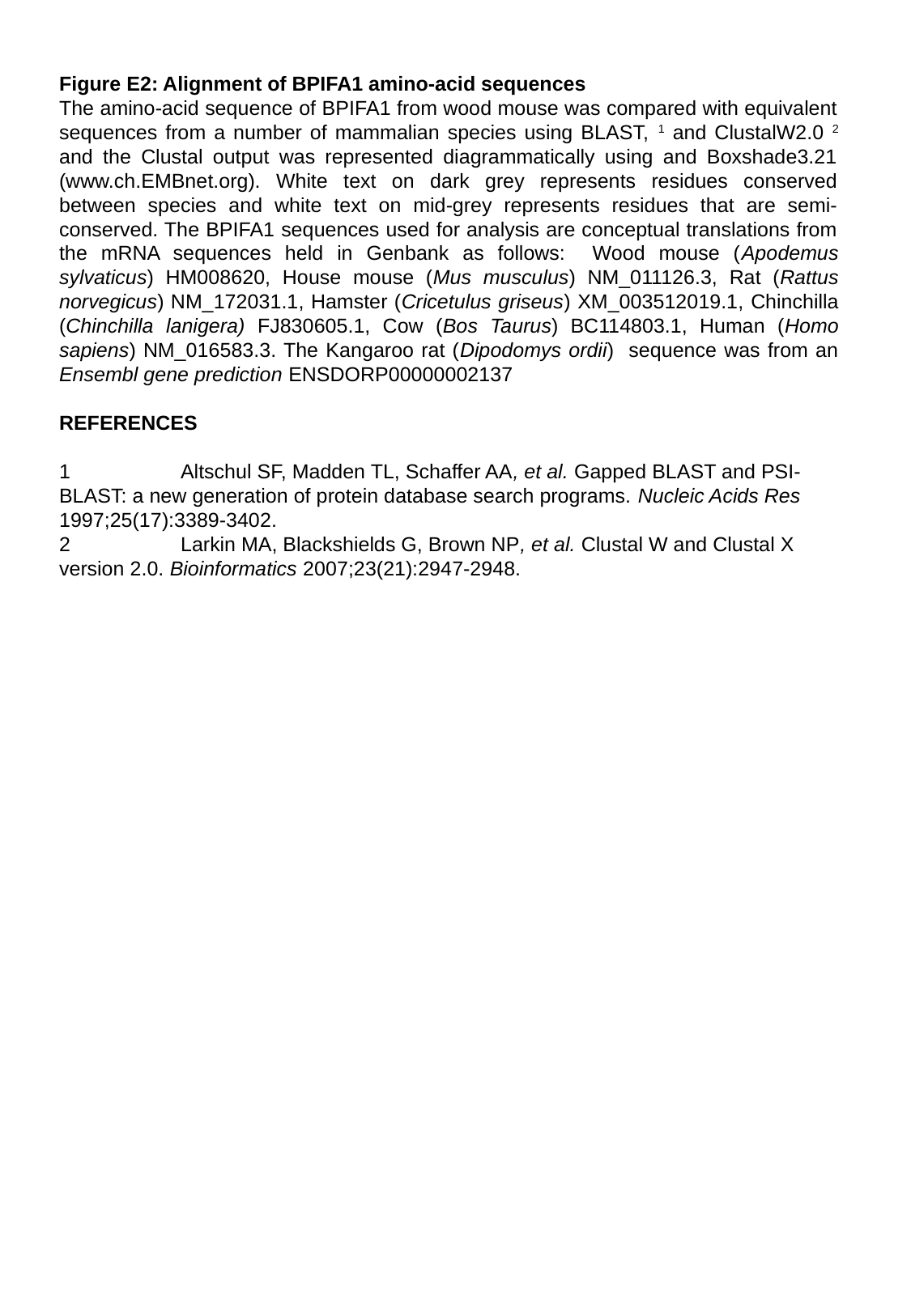

Figure E2: Alignment of BPIFA1 amino-acid sequences
The amino-acid sequence of BPIFA1 from wood mouse was compared with equivalent sequences from a number of mammalian species using BLAST, 1 and ClustalW2.0 2 and the Clustal output was represented diagrammatically using and Boxshade3.21 (www.ch.EMBnet.org). White text on dark grey represents residues conserved between species and white text on mid-grey represents residues that are semi-conserved. The BPIFA1 sequences used for analysis are conceptual translations from the mRNA sequences held in Genbank as follows: Wood mouse (Apodemus sylvaticus) HM008620, House mouse (Mus musculus) NM_011126.3, Rat (Rattus norvegicus) NM_172031.1, Hamster (Cricetulus griseus) XM_003512019.1, Chinchilla (Chinchilla lanigera) FJ830605.1, Cow (Bos Taurus) BC114803.1, Human (Homo sapiens) NM_016583.3. The Kangaroo rat (Dipodomys ordii) sequence was from an Ensembl gene prediction ENSDORP00000002137
REFERENCES
1	Altschul SF, Madden TL, Schaffer AA, et al. Gapped BLAST and PSI-BLAST: a new generation of protein database search programs. Nucleic Acids Res 1997;25(17):3389-3402.
2	Larkin MA, Blackshields G, Brown NP, et al. Clustal W and Clustal X version 2.0. Bioinformatics 2007;23(21):2947-2948.
